# Innovative Method for Coating of Natural Corrosion Inhibitor Based on *Artemisia vulgaris*

**DOI:** 10.3390/ma14092234

**Published:** 2021-04-26

**Authors:** Daniel Alejandro Pineda Hernández, Elisabeth Restrepo Parra, Pedro José Arango Arango, Belarmino Segura Giraldo, Carlos Daniel Acosta Medina

**Affiliations:** 1 Laboratorio de Física del Plasma, Unicversidad Nacional de Colombia sede Manizales, Manizales-Caldas 170003, Colombia; erestrepopa@unal.edu.co (E.R.P.); pjarangoa@unal.edu.co (P.J.A.A.); bsegurag@unal.edu.co (B.S.G.); 2 Calculo Científico y Modelamiento Matemático, Universidad Nacional de Colombia sede Manizales, Manizales-Caldas 170003, Colombia; cdacostam@unal.edu.co

**Keywords:** EIS, organic coating, tafel, mild steel, corrosion

## Abstract

In this work, the production of a novel methodology for the application of natural corrosion inhibitors on steel, using an autoclave is presented. Tests were carried out using *Artemisia vulgaris*. The inhibitor was produced with a simple soxhlet extraction process using 15 g of *Artemisia vulgaris* and 260 mL of Ether. Once the inhibitor was produced, the steel was immersed in it, to form a coating that protects the material against corrosion. Thermogravimetry analyzes (TGA) were performed on the inhibitor, to determine the degradation temperature; it was observed that, at 321 °C, the loss of organic mass begins. After applying the inhibitor to the steel, the Fourier Transform Infrared Spectroscopy (FTIR) technique was used to determine the vibrational bands and the difference between the spectra for the steels before and after the coating was applied. For the evaluation of the method efficiency, Electrochemical Impedance Spectroscopy (EIS) and polarization resistance tests were performed, where Nyquist diagrams and Tafel curves were obtained, for steels with and without treatment. In this case, an increase of 93% in the corrosion resistance, and an 88% decrease in the corrosion rate were observed, proving that this methodology can be used to protect steel against corrosion and extend the steel’s useful life.

## 1. Introduction

Each minute, 300 tons of steel are dissolved around the world due to corrosion, generating millions in losses for governments [1,2]. Due to this phenomenon, corrosion emerges as one of the biggest problems in the modern world, which makes it necessary to develop more efficient protection and prevention solutions against corrosion. To counter this problem, researchers have proposed solutions such as coatings [3], paints [4], thin films [5], corrosion inhibitors [6,7,8], among others; however, currently, one of the most important requirements for anti-corrosion solutions is that they should be as little polluting as possible.

In this field, corrosion inhibitors produced from natural sources are very promising.

Corrosion inhibitors are substances that, when added in small amounts to a corrosive medium, can decrease the rate of deterioration of the material through passivation [7]. There are several commercial corrosion inhibitors, which are widely used by the industry. These inhibitors are a useful tool in the battle against corrosion, as they reduce costs and improve the useful life of the material [8]. However, the composition of most of these corrosion inhibitors is unknown; besides, these corrosion inhibitors are toxic and environmentally harmful substances. In addition, they could be dangerous for the personnel who handle them. For this reason, in the last decade, governments have created regulations such as the Toxic Substances Control Act of the United States Environmental Protection Agency (EPA) and the European Union’s Restriction of Hazardous Substances Directive [9]. These regulations demand that the products used in the industrial field must have the minimum possible toxicity. In order to search for options to comply with these laws, researchers worldwide are proposing the use of plant, fruit and/or flower extracts as corrosion inhibitors [10].

The study of corrosion inhibitors from natural sources has been advanced through systematic studies of plants and fruits on different types of metals, especially steel due to its wide range of applications. Thanks to these studies, a new horizon has been discovered, since these substances are very efficient under different corrosive conditions, with superior ecological properties since they are biodegradable. For instance, N. Soltani and collaborators [7] studied the inhibitory character of Salvia Officinalis in austenitic stainless steel 304; H. Herrera-Hernández et al. [8] investigated aloe vera gel on structural reinforcing steel, finding that this type of inhibitors are highly efficient for the protection of steels in different corrosive medium with protection efficiencies greater than 80% in comparison with steels without inhibitor addition, and many reports show that this type of inhibitor increases the useful life of materials by 80–90% [11,12,13].

Specifically referring to *Artemisia vulgaris* as a corrosion inhibitor, in 2012, Subhadra Garai and collaborators, from the National Metallurgy Laboratory of Jamshedpur, India, studied the inhibitory character of this plant. In this work, it was determined that the methanoic extract of *Artemisia vulgaris* shows efficiencies of 93% in 1 mol L^−1^ HCl [6]. *Artemisia vulgaris* is a plant belonging to the Asteraceae family. Despite being considered undergrown, this family of plants has been studied extensively due to their antibacterial, antiseptic, and antioxidant properties. It grows in temperate climates and is native to Europe [14].

Generally, applying this type of corrosion inhibitors to the corrosive medium shows a considerable disadvantage at the application level against other types of solutions, such as paints or coatings. Unlike previous works related to corrosion inhibitors from natural sources, this work proposes an innovative, economical, and viable solution, which consists of the inhibitor adsorption by the metal using a hydrothermal process. The process generates optimal conditions for the creation of a natural extract layer that acts as a corrosion inhibitor. For an initial test, the work carried out by N. Soltani and collaborators was considered, since they used *Artemisia vulgaris* as a corrosion inhibitor for structural steel in a 1 mol L^−1^ HCl solution, and based on this work, the autoclave method was applied.

## 2. Materials and Methods

### 2.1. Extraction and Inhibitor Application

**Figure 1:**
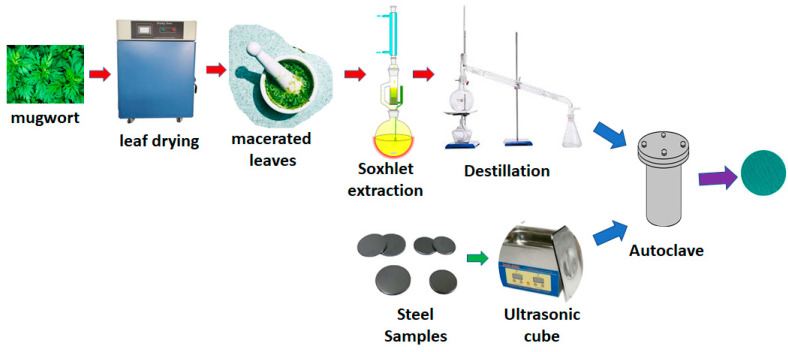
Experimental setup.

Figure 1 shows a diagram of the methodology used to extract the corrosion inhibitor from *Artemisia vulgaris,* and its application to the steel samples employing the autoclave. To carry out the extraction, leaves were taken from the plant and a drying process was carried out in a Humboldt MFG oven. The samples were in a Model 40 Go lab oven at 60 °C for 24 h, afterwards, they were macerated until a fine powder was produced [15]. A sample of 20 g of this powder was taken and using a Soxhlet, the extract was prepared in 260 mL of ether for 4 h. Afterward, the ether solution was distilled to concentrate the extract until 20 mL of solution was acquired [16]. After that, a thermogravimetric analysis of the extract was carried out, to determine the degradation temperature. The structural steel samples to be coated must be partially polished and cleaned with acetone to remove any dirt from the surface.

For optimal working conditions, a systematic study was conducted varying the temperature from 60 °C to 200 °C with a step of 20 °C in the hydrothermal process. It was observed that at low temperatures, the coating was not formed and at high temperatures the extract was calcined. The temperature at which the coating showed better characteristics was 120 °C; then, this temperature was selected for developing the process of coating the steel.

After the extract was obtained and the steel samples were thoroughly cleaned, they were put into the autoclave, and it was hermetically sealed and heated. This heating was carried out with a filling factor of 30%. In this filling factor, 20% of the volume corresponded to the extract and approximately 10% to the metal immersed in it. The heating was carried out for 40 min. At the end of this time, the sample was extracted, dried under normal environmental conditions, and the tests were subsequently carried out. To develop this experiment, a stainless-steel autoclave with a total volume of 100 mL was used; inside the autoclave, there is a Teflon container to prevent the reaction of external compounds during the creation of the coating. Figure 1 shows a diagram that describes the experimental process to make the coatings.

### 2.2. Materials Characterization

Fourier transform infrared spectroscopy (FTIR) was used to determine the functional groups in the *Artemisia vulgaris* extract and the coatings on the structural steel. For this analysis, a BRUKER alpha platinum equipment with an ATR platinum Diamond 1 accessory was used. The characterization was carried out with a resolution of 4 cm^−1^, 32 steps and a measurement range from 400 to 4000 cm^−1^; at the same time, a thermogravimetric analysis using a TGA Q500 V6.7 Build 203 was performed to determine the degradation temperature of the extract. A heating rate of 293.15 Kmin^−1^ was applied until reaching 800 °C. A nitrogen atmosphere was used with a flow of 60 mL min^−1^. To evaluate the efficiency of the coating, electrochemical impedance, and polarization resistance spectroscopy was performed to determine corrosion resistance and corrosion rate using a Gamry 1000E potentiostat/galvanostat. EIS tests were performed in a range of 10^6^ to 10^−3^ Hz employing 0.1 mol L^−1^ HCl as a corrosive medium, while TAFEL tests were performed according to ASTM G59 recommendations [17].

## 3. Results

### 3.1. Compositional Characterization

#### 3.1.1. Fourier Transform Infrared

**Figure 2:**
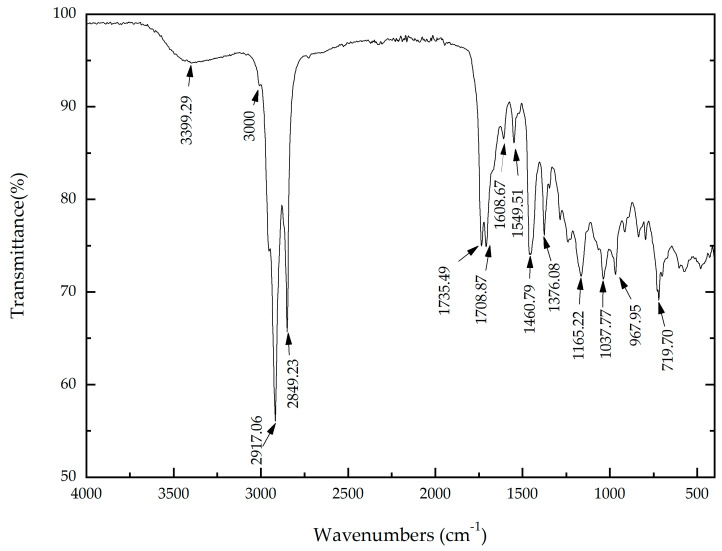
FTIR transmittance spectrum for *Artemisia vulgaris* extract.

Figure 2 shows the spectrum for the concentrated extract. A large number of CO, CH and CC bonds, characteristic of organic substances, was observed. The results are listed in Table 1 [18,19].

**Figure 3:**
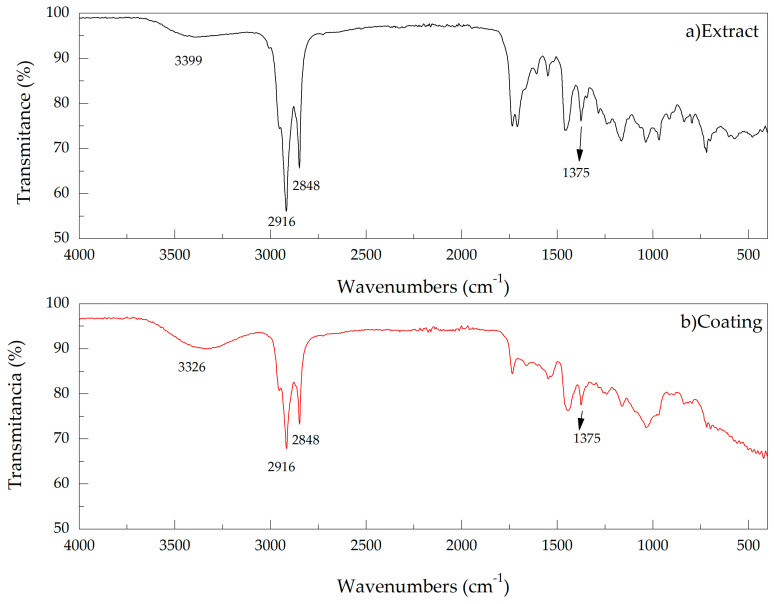
FTIR transmittance spectrum for (**a**) *Artemisia vulgaris* extract (**b**) coating of *Artemisia vulgaris.*

Figure 3 shows the comparison between the spectra for the coating, and the extract. It is possible to identify the functional groups in each of the cases. The conservation of most of the compounds in the steel coated according to the extract can be observed. This is because the steel adsorbed several substances from the extract. In both cases, some bands correspond to hydroxy-phenolic groups (3336.4 cm^−1^), aromatic groups (1653.94 cm^−1^), groups that, according to the literature, are the ones that improve corrosion inhibition [6,20,21,22]. On the other hand, changes in the intensities of some peaks of the extract were observed once applied to the steel. This effect is because the steel adsorption of species is not total and therefore, the concentration of each substance is lower. The preservation of the compounds that inhibit corrosion on the surface of the steel coating is because a thermogravimetric analysis was previously carried out to find the temperature at which the extract begins to degrade. Once the degradation temperature was known, the coating process was carried out at a temperature lower than the one found in the thermogravimetric analysis.

#### 3.1.2. Thermogravimetric Analysis

**Figure 4:**
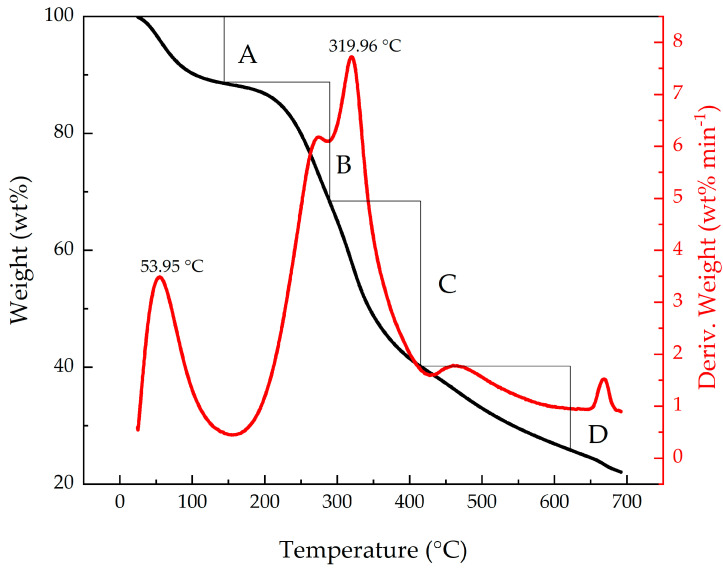
Thermal Decomposition Curve of the corrosion inhibitor produced from the *Artemisia vulgaris*.

In Figure 4, the thermal decomposition curve obtained by the thermogravimetric analysis of the *Artemisia vulgaris* extract is presented. The decomposition of the extract as a function of temperature can be observed, showing critical points of mass loss. It can be deduced that the first significant loss of mass, corresponding to 20.25 wt% of the total, begins at 52.2 °C with the surface water and ends between 100–105 °C with bound water [23]. Subsequently, at higher temperatures, between 125–350 °C, 29.22% of the mass is lost, corresponding to the C=O bonds and the decomposition of the OH groups [11,18].

Table 2 shows in detail the loss of mass with the increase of temperature, however, since in this investigation it is required for the extract to remain as intact as possible, the working temperature must be lower than the first critical point of mass loss, that is, less than 325 °C. For this reason, it was decided to coat the steel with the extract inside the autoclave, at a temperature of 120 °C, at which the compound has not been degraded.

### 3.2. Electrochemical Measurements

**Figure 5:**
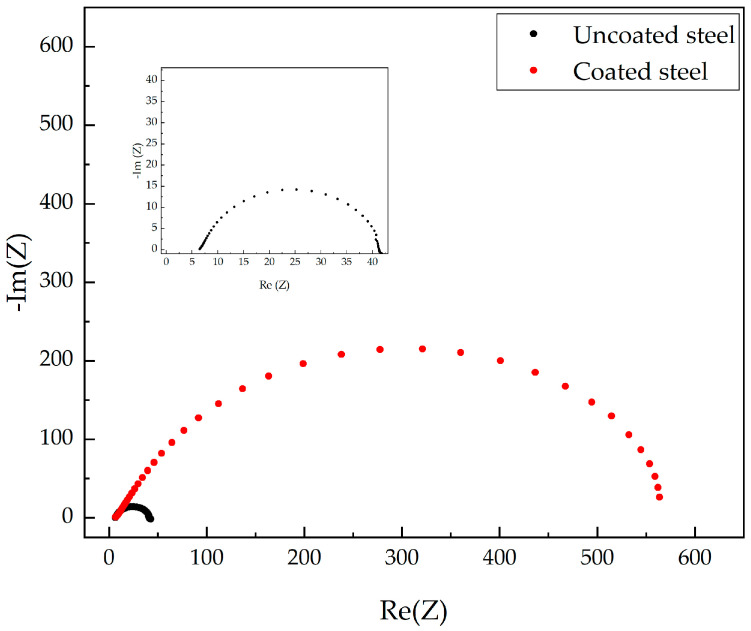
Nyquist plot of mild steel in 0.1 mol L^−1^ HCl with and without the coating of *Artemisia vulgaris* extract.

Figure 5 shows the Nyquist diagram from the electrochemical impedance spectroscopy technique. The black and red curves correspond to uncoated and coated steel samples, respectively. From this figure, it is observed that the coated steel exhibits a much greater radius of curvature than the uncoated steel, which indicates a noticeable increase in the corrosion resistance. The efficiency of the coating was calculated using Equation (1), where % ef is the coating efficiency, SS and SR the corrosion resistance of the steel and the coated steel, respectively, [24,25,26,27], finding an increase of 92.91% in the corrosion resistance, as shown in Table 3.

(1) $$\%ef=\frac{SS-SR}{SR}*100\%$$

**Figure 6:**
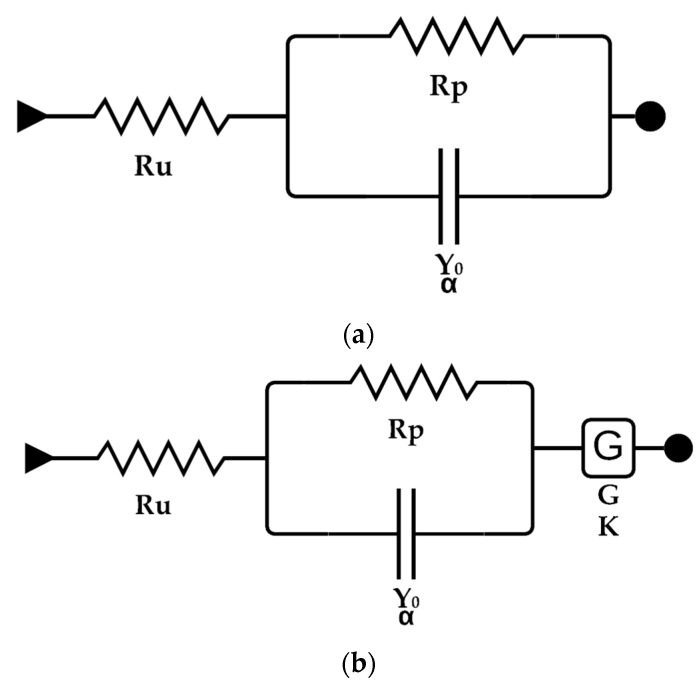
Equivalent circuits for (**a**) uncoated steel (**b**) coated steel.

The behavior of treated and untreated steel can be modeled from electronic components to understand better the electrical behavior of the corrosion process. The equivalent circuits for untreated and treated steel, respectively, are shown in Figure 6a,b.

Figure 6 shows the different components that model the electrochemical behavior of the structural steel surface with and without treatment. R_u_ is related to the resistance of the aqueous solution. Y_0_ is a constant phase element associated with the electronic double layer capacitance, created on the interface surface of the working and solution electrode; however, since it is not an ideal capacitance due to roughness and possible pores on the surface of the steel, with an ideality factor α, the gerischer element G, that is found only in the equivalent circuit of the treated steel, is an indication of the porosity of the organic coating with a porosity factor K, the gerischer element is necessary to explain the reaction of the organic coating, because the reactions between the two surfaces, the surface of the steel and the organic coating, cannot be distinguished from each other [28]. Finally, R_p_ is the polarization resistance or corrosion resistance of the material [27,29,30]. This variable is the one of interest for the study. Table 3 and Table 4 show the values of these elements.

**Figure 7:**
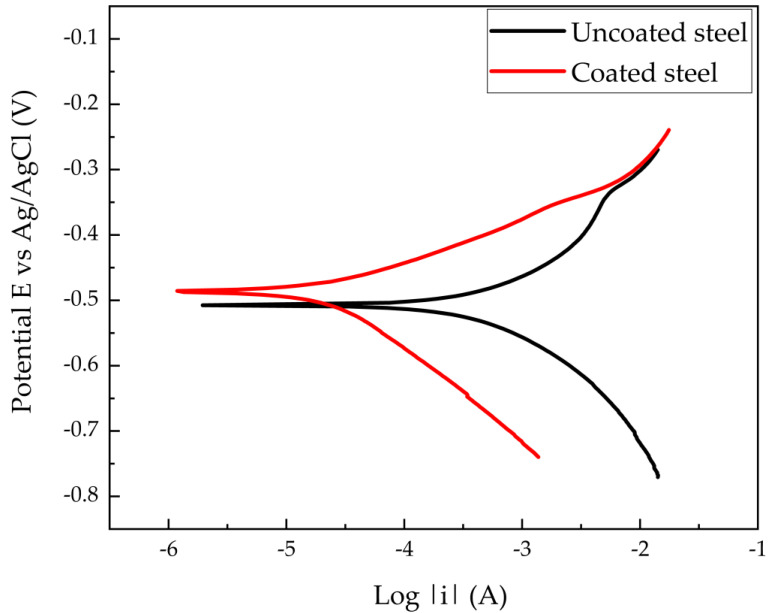
Potentiodynamic curves of mild steel in 0.1 mol L^-1^ HCl with and without coating of *Artemisia vulgaris* extract.

By using the potentiodynamic curves, different data can be obtained regarding the phenomena occurring in the corrosion and inhibition processes once the extract is applied to the Steel. In addition to obtaining information on the adsorption of the inhibitor employing an autoclave. Figure 7 shows the Tafel polarization curves corresponding to uncoated and coated steel samples. This figure shows an inhibitory behavior of an anodic nature since it moves towards the anodic part of the curve. This anodic inhibition behavior is related to the formation of films on the steel surface due to external printed currents [31,32]. Furthermore, it is observed that the corrosion rate is reduced by 32.6%, with respect to the corrosion rate of the untreated steel. This decrease is because the inhibitor generates an oxidoreduction reaction process, delaying the release of ions from the Steel, which is proof of the efficiency of the coating. Table 5 show the corrosion resistances (Rcorr) and corrosion rate of uncoated and coated steel with the efficiency shown for each parameter.

### 3.3. Visual Inspection

**Figure 8:**
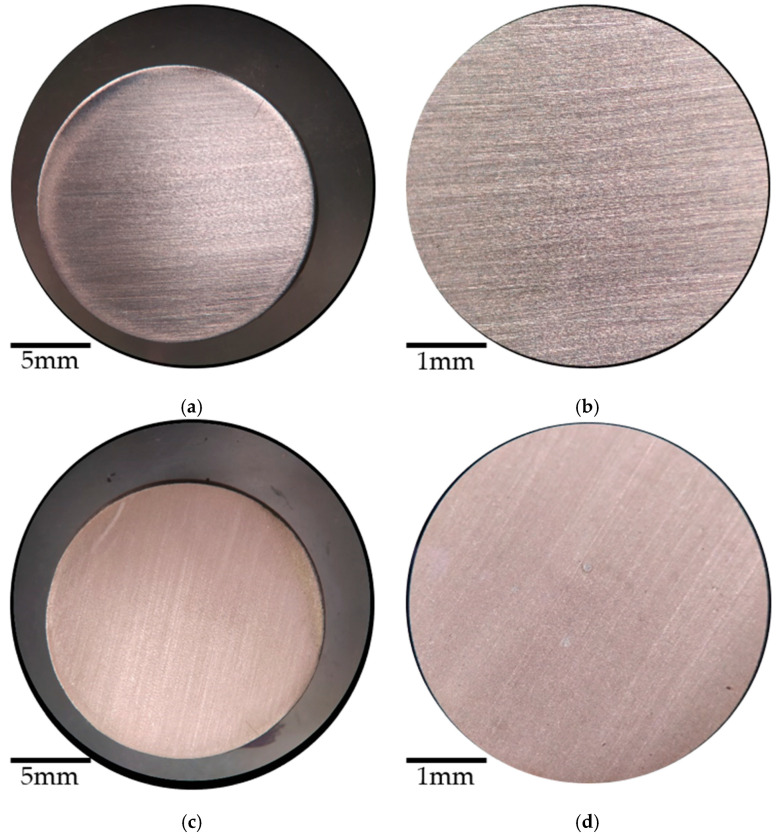
Micrographs before corrosion of structural steel (**a**) polished with magnification 1×, (**b**) polished with magnification 5×, (**c**) coated with magnification 1×, and (**d**) coated with magnification 5×.

Figure 8 shows the micrographs of the structural steel samples with and without coating before the corrosion measurements. Figure 8a,b show the polished structural steel with the characteristic shine of the steel. In Figure 8c,d, the steel coated with *Artemisia vulgaris* is presented. When these two samples are compared, a difference in their brightness is observed, the sample in Figure 8c,d shows an opacity in addition to a jade green pigmentation developed due to the coating.

**Figure 9:**
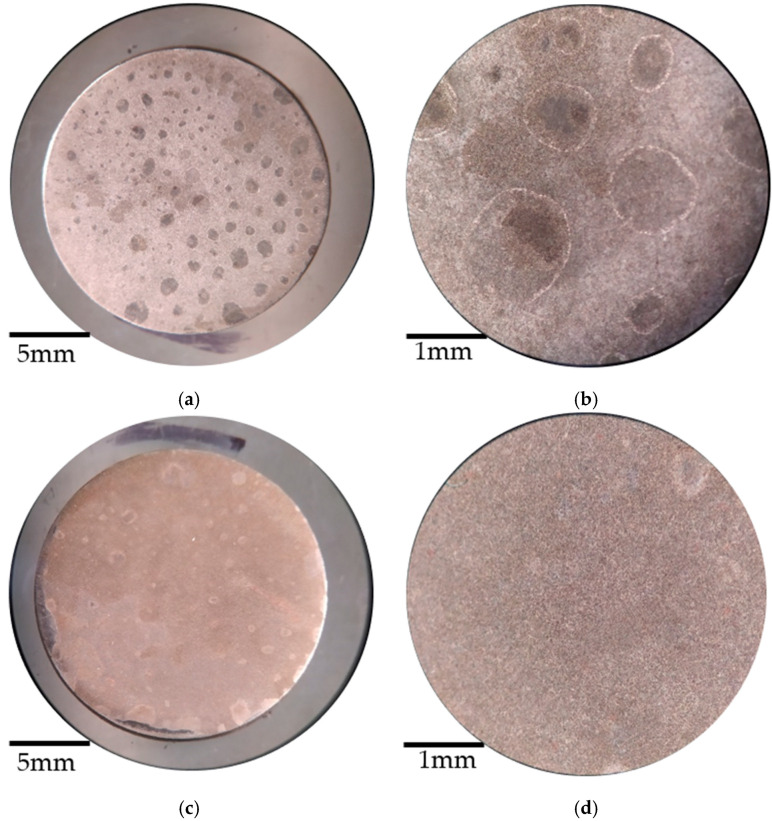
Micrographs after corrosion measurements of structural steel (**a**) polished with magnification 1×, (**b**) polished with magnification 5×, (**c**) coated with magnification 1×, and (**d**) coated with magnification 5×.

Figure 9 shows the micrographs corresponding to the samples after the corrosion measurements. Figure 9a shows the corrosion of the mild steel sample, where localized sources of corrosion are present. When these corrosion points are seen more closely in Figure 9b, characteristic pitting corrosion is evident in this type of steel. On the other hand, in Figure 9c,d it is observed that the sample coated with *Artemisia vulgaris* changed its color from jade green to an opaque brown, as a consequence of the reaction between the phenols and flavonoids of the coating with the medium corrosive [33].

**Figure 10:**
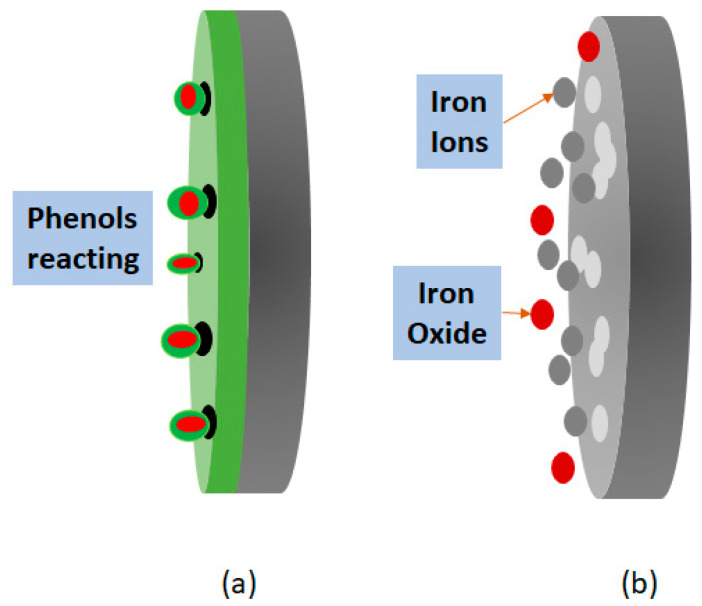
Schematic description of the phenomenology occurring for the prevention of deterioration by coating with natural corrosion inhibitors; (**a**) corrosion phenomenology with coating; (**b**) corrosion phenomenology without coating.

Figure 10 shows the coating inhibition method. The protection provided by *Artemisia vulgaris* coating is possible thanks to the ability of phenols to trap oxygen and hydrogen in their free radicals [33,34], phenols being part of the oxidation process while the steel is protected.

## 4. Conclusions

A natural corrosion inhibitor coating was obtained from the *Artemisia vulgaris* plant using the autoclave method. TGA analyses showed that the material begins its degradation at 325 °C. Then, lower temperatures must be used. Using FTIR, the vibrational bands in the coating and the *Artemisia vulgaris* extract were determined, observing hydroxy-phenolic (3336.4 cm^−1^) and aromatic (1653.94 cm^−1^) groups that, according to the literature, help the inhibitory character of these substances. Corrosion resistance was determined using Nyquist diagrams, where a 59.95% of increase in corrosion resistance was obtained. It is important to highlight that there is good adsorption of the inhibitor by the steel; this can be evidenced in the FTIR analyzes and in the corrosion tests, specifically in EIS, where the reaction of the coating and the substrate with the medium cannot be differentiated. In addition, it was determined that the corrosion current decreased and the voltage increased, which is an indication of an increase in the useful life of the steel, showing an improvement in the corrosion rate of 88.31%.

This method was innovative and effective in protecting structural steels since it provides a homogeneous coating of the surface exposed to the treatment and should be studied to obtain a better understanding of the adsorption mechanisms.

## Figures and Tables

**Table 1:** FTIR spectrum identification for *Artemisia vulgaris* extract.

Wavenumber (cm^−1^)	Compound	Notes
3399.29	OH	Associated with a polymeric primary alcohol (carrier substance)
3000	-CH_2_-	Vs
2917.06		Vas
2849.23		Vs
1460.79		Scissor
719.70		Rocking
1376.08	O-CO-CH_3_	
1549.51	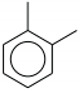	Confirmed presence of aromatic rings with benzene nucleus.
1608.67		
1165.22		They give information about the branches that the ring presents.
1037.77		
967.94		
1735.49	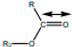	Esters
1708.87	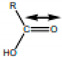	Carboxylic acids

**Table 2:** Mass loss percentage.

	% Weight	Temperature (°C)
A	11.38	52.24
B	20.25	288.28
C	29.22	321.64
D	13.07	618.45

**Table 3:** Value of equivalent circuit components for uncoated steel.

Element	Value	±Error	Units
**R_u_**	6.607	40.05 × 10^−5^	Ω
**Y_0_**	454.4 × 10^−6^	19.95 × 10^−6^	S × s^α^
**α**	841.6 × 10^−3^	7.136 × 10^−3^	-
**R_p_**	35.53	289.7 × 10^−3^	Ω

**Table 4:** Value of equivalent circuit components for coated steel.

Element	Value	±Error	Units
**R_u_**	6.101	97.19 × 10^−3^	Ω
**Y_0_**	59.29 × 10^−6^	5.415 × 10^−6^	S × s^α^
**α**	890 × 10^−3^	12.38 × 10^−3^	-
**G**	1232 × 10^−3^	55.50 × 10^−6^	S × s^(½)^
**K**	181.5	77.64	s^−1^
**R_p_**	501.2	13.24	Ω

**Table 5:** Results from the electrochemical tests.

Sample\Charact.	Uncoated Steel	Coated Steel	%Efficiency
**Rcorr (Ω)**	35.53 ± 289.7 × 10^−3^	501.2 ± 13.24	92.91%
**Corrosion Rate (mpy)**	52.3	6.34	88.31%

## Data Availability

Data available on request due to restrictions eg privacy or ethical The data presented in this study are available on request from the corresponding author. The data are not publicly available due to these results are associated with an active project at the National University of Colombia.
